# Improved general regression network for protein domain boundary prediction

**DOI:** 10.1186/1471-2105-9-S1-S12

**Published:** 2008-02-13

**Authors:** Paul D Yoo, Abdur R Sikder, Bing Bing Zhou, Albert Y Zomaya

**Affiliations:** 1 Advanced Networks Research Group, School of Information Technologies (J12), The University of Sydney, NSW 2006, Australia; 2 Biotech Research Center, Michigan Technological University, Houghton, MI, 49931, USA; 3 Sydney Bioinformatics Centre and the Centre for Mathematical Biology, The University of Sydney, Sydney, NSW 2006, Australia

## Abstract

**Background:**

Protein domains present some of the most useful information that can be used to understand protein structure and functions. Recent research on protein domain boundary prediction has been mainly based on widely known machine learning techniques, such as Artificial Neural Networks and Support Vector Machines. In this study, we propose a new machine learning model (IGRN) that can achieve accurate and reliable classification, with significantly reduced computations. The IGRN was trained using a PSSM (Position Specific Scoring Matrix), secondary structure, solvent accessibility information and inter-domain linker index to detect possible domain boundaries for a target sequence.

**Results:**

The proposed model achieved average prediction accuracy of 67% on the Benchmark_2 dataset for domain boundary identification in multi-domains proteins and showed superior predictive performance and generalisation ability among the most widely used neural network models. With the CASP7 benchmark dataset, it also demonstrated comparable performance to existing domain boundary predictors such as DOMpro, DomPred, DomSSEA, DomCut and DomainDiscovery with 70.10% prediction accuracy.

**Conclusion:**

The performance of proposed model has been compared favourably to the performance of other existing machine learning based methods as well as widely known domain boundary predictors on two benchmark datasets and excels in the identification of domain boundaries in terms of model bias, generalisation and computational requirements.

## Background

Since proteins provide some of the most fundamental information about many processes in almost all organisms, the ability to predict protein structure and function has become one of the most important goals in bioinformatics research [1,2]. Protein domains represent one of the most useful avenues for the understanding of protein function and domain family-based analysis, and are of great importance in the study of individual proteins [3]. They have been described as units of compact structure [4], function and evolution [5] and folding [6]. In nature, several domains can be formed together with a vast number of possibilities leading to multi-domain and multi-functional proteins [7]. Each domain in a multi-domain protein has its own functions and jointly works with its neighbours. They serve as modules to form large assemblies like viral particles or provide specific catalytic and binding sites such as those found in enzymes or regulatory proteins.

Over the last three decade, a large number of methods using the three dimensional coordinates of the protein structure have been proposed for more accurate delineation of domains boundaries [8]. Recently, several methods developed to identify domains in globular proteins from one dimensional atomic coordinates and have been gaining much attention. These methods are based on the assumption that a domain has relatively more contacts within itself than with residues in the remainder of the structure [9]. The most recent sequence based methods have been built on the basis of various machine learners.

DOMpro [10] uses evolutionary information (gene-exon shuffling), secondary structure and solvent accessibility information with a recursive neural network; CHOPnet [3] utilises evolutionary information, amino acid composition and amino acid flexibility analysed with a neural network; SnapDRAGON [11] predicts domain by using an *ab initio *protein folding method; DomSSEA [12] uses predicted secondary structure; the Nagarajan and Yona's [13] method is based on analysing multiple sequence alignments from database searches, position specific physio-chemical properties of amino acids and predicted secondary structures analysed with a neural network; Galzitskaya and Melnik [9] use side chain entropy of a region to predict domain boundaries; SSEPDomain [14] predicts domains by combining information of secondary structure element alignments, profile-profile alignments and pattern searches; Armidillo [15] uses the amino acids composition to predict domain boundaries; DomCut [16] uses a linker index deduced from a set of domain/linker segments; PRODO [17] uses a neural network method and finally DomainDiscovery [18] uses support vector machines from sequence information including domain linker index. Many of these methods focus exclusively on predicting boundaries for two-domain chains. The overall accuracy of sequence-based methods has been reported in the range of 50 to 70% for single domain proteins and considerably less (<40%) when limited to multi-domain proteins [19,20].

Although a large number of machine learning based methods have showed their superior performance in domain boundary prediction, several important issues that can degrade the performance of machine learning or statistical-based methods have largely been ignored. It has been widely recognised that high dimensionality of protein sequence data not only causes a dynamic increase in computational complexity but also can be induced into the generalisation problem of non-parametric methods. With machine learning models, better generalisation and faster training (computationally efficient) can be achieved when they have fewer weights to be adjusted by fewer inputs.

This study aims to develop an accurate and reliable protein domain boundary prediction model using state-of-the-art machine learning techniques. In this paper, we propose a new semi-parametric method where a linear dimensionality reduction method and a non-parametric model are integrated, and show great performance in terms of prediction bias, variance and computational requirements. In the prediction, we use inter domain linker regions with position-specific scoring matrix (PSSM) generated from PSI-BLAST [21], secondary structural information and relative solvent accessibility data. Domain linkers have shown to be useful in cooperative inter-domain interactions [22] function regulation [23], protein stability [24], folding rates [25] and domain-domain orientation [26]. The others were adopted based on the assumption that secondary structure elements and the level of solvent accessibility in the boundary regions are different from those found in the rest of the protein. The novel features of this research are the use of a new semi-parametric model and importantly, a unique training set (Benchmark_2) built on the consensus of various experts in protein structure. In the literature, as the word "protein domain" is used in various contexts, we adopt the CASP7 definition of protein domain in this study. Domains in CASP7 should fall somewhere in the middle of following three definitions. (1) Conserved structural entities with a hydrophobic core. (2) Regions that share a common fold, have some functional similarity, and may be evolutionarily related. (3) A subunit of a polypeptide chain that can fold into a stable tertiary structure independently of any other domain. However, predictors are only able to evaluate predictions made for the first two definitions.

## Results

A fair and precise comparison of domain boundary predictors is complicated by the fact that the existence of various domain datasets/databases which may conflict and are biased in some cases [27]. For a fair comparison of the proposed model to other current methods, we have tested each model with Benchmark_2 dataset, a new comprehensive dataset that was developed for the purpose of benchmarking structure-based domain identification methods [28] and CASP7 dataset, the most widely known benchmark in protein structure prediction.

The objectives of this study are two-fold. The first is to show the effectiveness of the proposed model by accurately comparing it with state-of-the-art machine learning models in terms of prediction ability, stableness/robustness, and computational efficiency. At the same time, we find the most suitable window size for the given datasets. Second, by using the best window size, we compare the predictive performance of the proposed model with existing and widely known domain boundary predictors on CASP7 benchmark dataset.

With the first objective, we use the Benchmark_2 dataset and adopt a seven fold cross-validation scheme for our model evaluation. We evaluate the performance of each model by looking at the average prediction accuracy, the variance of the accuracy, the average training and testing time obtained over our four different datasets constructed based on different window sizes (win_7, win_11, win_19 and win_27). The performance of our proposed model are compared with other general Artificial Neural Network (ANN) models such as Multi-Layered Perceptron (MLP), Radial Basis Function Network (RBFN) and General Regression Neural Network (GRNN).

**Figure 1:**
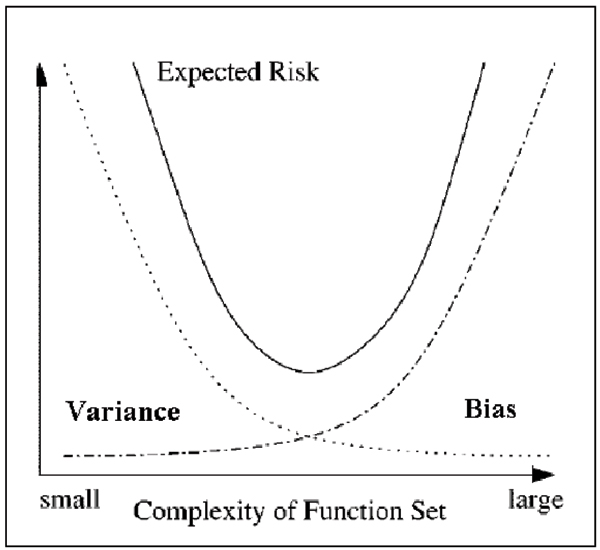
Bias and variance dilemma of parametric and non-parametric models.

In order to validate the effectiveness of our proposed model, the model should be able to achieve a good trade-off between the model bias and generalisation variance (Figure 1). Table 1 presents the average prediction accuracy, the variance of prediction accuracy of each prediction model on the seven testing samples for four different window sizes as well as the average training and testing time of each model. As observed, IGRN produced the comparable predictive performance, the overall prediction accuracy of 64.79% and the smallest variance (0.86) than that of GRNN, RBFN and MLP. In the experiment results above, the semi-parametric approach of IGRN was demonstrated to be effective for stable prediction. Although our semi-parametric approach (IGRN), the incorporation of reduced dimension of input vectors into pure non-parametric model has brought better generalisation and less computational requirements, the prediction accuracy of the proposed model is slightly less accurate (64.79%) than original model, GRNN (65.36%) on the Benchmark_2 dataset.

**Table 1:** The prediction accuracy, generalisation variance and computational efficiency of machine learning models.

	IGRN II	IGRN	GRNN	RBFN	MLP
Win 7	68.3	65.6	67.1	65.4	66.7
Win 11	66.7	65.4	65.1	61.3	63.8
Win 19	67.1	63.7	65.4	62.1	66.5
Win 27	65.5	64.5	63.8	59.7	62.1
Avg Accuracy	67.0	64.79	65.36	62.13	64.78
St. Dev	1.16	0.86	1.37	2.41	2.22
Training Time	43.2	31.5	91.3	28.1	91.8
Testing Time	7.4	5.3	8.7	5.2	6.3

In order to reduce the learning bias of the IGRN, we adopted a boosting algorithm into the IGRN and it eventually showed much better trade-off between learning bias and generalisation variance that that of IGRN. IGRN II (AdaBoost embedded in IGRN) achieved the best prediction accuracy as well as comparably low variance among different neural network models. As for the computational efficiency, IGRN and IGRN II both showed comparable performance over other three machine learners. It means our semi-parametric approach of proposed model not only has more stable prediction capability than other ANN models but also demands less computation requirements.

In Table 1, we can observe that the highest accuracy was found in the window size of seven for all models and gradually became less as the size of window got longer. This is in some way an expected result as amino acid residues residing closer to the linker residue contain more structural information which involves stronger short-range interaction signals than others.

With the result obtained from above experiment, the predictive performance of the proposed model is now compared with the most widely known domain boundary predictors, namely, DOMpro, DomPred, DomSSEA, DomCut and DomainDiscovery. DOMpro [10] uses machine learning algorithms, in the form of recursive neural networks and predicts protein domains using a combination of evolutionary information in the form of profiles, predicted secondary structure, and predicted relative solvent accessibility. DomPred uses a combined homology and fold recognition-based approach. The sequence homology approach simply attempts to distinguish domain boundaries from overlapping edges in PSI-BLAST multiple sequence alignments. The fold recognition approach relies on secondary structure element alignments, using the DomSSEA method [12], in order to find domain boundaries in more distant homologs. The method has an accuracy of 49% at predicting the domain boundary location within 20 residues using a representative set of two domain chains. DomCut [16] predicts linker regions based on sequence alone, relies solely on amino acid propensity. This method simply defines a linker region to be one that has lower linker index values than a specified threshold value. The linker index of amino acids are computed from a database of about 235 non-redundant (pairwise homology <30%) protein sequences. Only the continuous domain (functional or structural) segments of size 50 to 500 are chosen provided the linker region between them is of the size 10 to 100 residues. Linker index of an amino acid is the log ratio of its frequency of being present in a linker region and the frequency presence in a domain region. As used in DomCut, IGRN will also employ domain linker index to model linker regions. Finally, DomainDiscovery [2] is newly developed machine learning based domain predictor. It uses Support Vector Machines (SVMs) and additional sequence-based information, domain linker index during its training. It has shown its comparable predictive performance to other widely known domain predictors in the prediction of domain boundary.

Table 2 shows the experiment results of five domain boundary predictors and our proposed models on CASP7 dataset. The dataset contains 64 1-domain chains, 21 2-domain chains and 2 3-domain chains. IGRN models have shown the comparable performance to other domain boundary predictors as well as its original model (GRNN). IGRN II showed the best prediction accuracy amongst the state-of-the-art predictors by reaching the prediction accuracy of 69.44%. IGRN II correctly predicted at 87.93%, 19.12%, and 7.50% accuracy for 1-domain, 2-domain, and 3-domain chains respectively. Interestingly, although the overall accuracy of IGRN (68.10%) is slightly less than that of GRNN (68.52%), its performance for 2-domain and 3-domain was about 7 points higher than that of GRNN. It may indicate that the semi-parametric approach of IGRN which brings more stable prediction seems more suitable for domain boundary prediction problem. In addition, IGRN II also showed superior performance on multi-domain chains. The stability/generalisation ability of our proposed models was again proved with the CASP7 dataset. However, it is noted that the prediction results for the 3-domain chains are not statistically significant as the CASP7 dataset contains only two 3-domain protein chains. The prediction performance of the proposed model (IGRN) should be further tested on more 3-domain or larger protein chains.

**Table 2:** Accuracy of domain boundary placement on CASP7 benchmark dataset.

	1-domain	2-domains	3-domains	Overall Accuracy (%)
DOMpro	84.4	0.0	0.0	62.64
DomPred	85.9	9.5	33.0	66.28
DomSSEA	80.5	19.1	33.0	62.06
DomCut	85.3	9.5	7.5	65.21
DomainDiscovery	80.5	31.0	29.2	67.34
IGRN II	87.9	19.1	7.5	69.44
IGRN	85.2	21.9	7.5	68.10
GRNN	88.3	14.8	0.0	68.52

**Figure 2:**
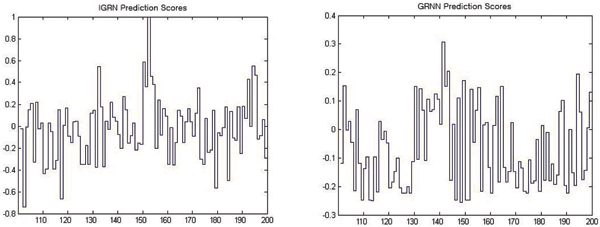
Comparison of domain boundary prediction scores between IGRN and GRNN. Domain Boundary is at the residue 155.

Figure 2 shows that the comparison of prediction scores simulated by IGRN and GRNN on a protein chain, CASP7 target number: T0318 (PDB code: 2HB6). The protein chain has two domains and its boundary is at the residue 155. IGRN gives clear indication of the domain boundary at the residue 155 and its signal is far stronger than that of GRNN by reaching almost 1 point (0.9964). However, GRNN's signal at the domain boundary is generally only around at 0.3 point which may be indistinguishable from neighbouring signals. Evidently, IGRN offers an additional level of advantages over GRNN and other existing predictors by providing more clear and strong indication of boundary location.

## Discussion

Although various machine learning based domain predictors have been developed, their limited stability/generalisation ability has revealed their inadequacy for domain boundary prediction. Our proposed semi-parametric approach was seemed to provide a good trade-off with both low bias and low variance. The experimental results confirmed our hypothesis that the IGRN produces improved generalisation while it is preserving the accurate data modelling in domain boundary prediction. However, as the prediction accuracy of our proposed model was slightly less (64.79%, IGRN) than that of original model (65.36%, GRNN) on two benchmark datasets, this issue should be taken into account.

From our extensive analysis, it may be caused by the linear nature of the embedded dimensionality reduction method, Principal Components Analysis (PCA). Most of the classical methods perform a linear transformation of the original feature vectors. PCA is the most widely known linear feature extraction techniques. However, it has been shown that its effectiveness is limited by the fact that it is globally linear methods. In other words, the data sets may contain essential non-linear structures that are invisible to PCA. It is generally believed that protein sequence data contains non-linear relationships which may be generated from long-range interactions. Thus, a new IGRN incorporated with an efficient non-linear dimensionality reduction method should be tested in the prediction of domain boundary.

One issue with current protein encoding methods used as input to the predictors is that they may provide insufficient structural information involving various interactions (short, middle and long-range interactions of protein). The formation of protein secondary structure, especially the regions of *β*-sheets, involves long-range interactions between amino acids [29]. With current methods, the prediction accuracy for *β*-strands is less than that for *α*-helix or coil. The *β*-sheet formation is seen as a tertiary structure interaction which brings two or more strands together by hydrogen bonds. However, they can be situated far apart in the amino acids sequence. In this case, the residues that are close in three dimensional space occupy distant positions in one dimensional sequence [30]. For statistical or machine learners, it is therefore crucial that the model should be further modified or tuned up to efficiently tackle the problem of long-range interaction or an efficient sequence encoding method that effectively represents various interactions should be developed for more accurate prediction of protein domain boundary.

Another considerable issue is that in general the prediction accuracy of sequence-based methods has been far less (<40%) for multi-domain proteins. For example, Liu and Rost's [27] experiments on CATH and SCOP assigned domains to random subsets of 1187 proteins of known high-resolution structure and less than 10% sequence homology, showed correct prediction of the number of domains (single or multi) in 69% cases. This accuracy for multi-domain case alone was however, only 38%. For the two-continuous domains proteins, average-accuracy of *dbp *prediction in different validation runs is 46% to 51% considering a prediction to be correct if it were in ±20 residues interval of the CATH and SCOP assigned boundaries. Our experiments on CASP7 dataset showed corresponding results as above.

Joshi [20] discussed the main reasons for the problems in deciphering the multi-domain protein structures and its possible solutions. With experimental data, although the structure within a domain is fixed, the relative positioning of two domains within the same chain can vary. For this reason, and for the fact that protein structural domains are independent folding units, it is unusual to find single crystal structures containing more than one domain. For similar reasons, protein modelling by database searching, sequence alignment and/or phylogenic analysis and etc is also better performed on a single domain, rather than a multi-domain polypeptide. Hence, in most cases, the number of domain in a protein should be firstly identified to determine the locations of such domains on the primary chain before embarking on any standard method of protein structure/function determination. The identification of linker regions connecting two distinct domains is also useful in finding domain boundary locations accordingly, several domain boundary predictors such as DomCut and DomainDiscovery employing domain linker information showed reasonably better predictive performance in domain boundary prediction.

## Conclusion

This paper identified the effectiveness and utility of the newly proposed machine learning model, namely Improved General Regression Network (IGRN) for protein domain boundary prediction. For a given set of high dimensional protein data, the combination of a linear principal component local model with a non-parametric global model provided a way of fine-tuning the model by the adjustment of a single smoothing parameter *σ *as well as providing efficient semi-parametric approximation. This was demonstrated by the two consecutive experiments. The semi-parametric approach used in IGRN was shown to be effective by finding an optimal trade-off between parametric and non-parametric models with less computational requirements. Finally, with the CASP7 benchmark dataset, IGRN II achieved the best prediction accuracy amongst the contemporary domain boundary predictors by showing its usefulness especially in multi-domain chains. However, as the prediction accuracy of IGRN is still limited to less than 70% for multi-domain protein datasets, there are still large rooms for further improvement. As discussed, the mid and long-term dependencies of amino acids play key roles for more accurate delineation of domain boundaries. Hence, further research will be carried out on the development of an efficient algorithm to rapidly sift through huge amounts of protein data with the strong capability to capture the mid and long-term information that reside in amino acids.

## Methods

### Datasets

In this study, we use two benchmark datasets, namely Benchmark_2 and CASP7. Benchmark_2 is a newly developed comprehensive dataset for the purpose of benchmarking structure-based domain identification methods. The Benchmark_2 is similar to the dataset published by Holland et al. [28]. It contains proteins of known structure for which three methods (CATH [31], SCOP [32] and literature) agree on the assignment of the number of domains. The dataset comprises of 315 polypeptide chains, 106 1-domain chains, 140 2-domain chains, 54 3-domain chains, 8 4-domain chains, 5 5-domain chains and 2 6-domain chains. The dataset is non-redundant in a structural sense: each combination of topologies occurs only once per dataset. Sequences of protein chains are taken from the Protein Data Bank (PDB) [33]. Secondary structure information and solvent accessibility are predicted for each chain in the Benchmark_2 using SSpro [34] and ACCpro [35]. Evolutionary information for each chain is obtained by Position Specific Scoring Matrix (PSSM), which was constructed using PSI-BLAST [21]. The inter-domain linker index was taken from DomCut [16].

CASP7 is one of the most widely known benchmark dataset in domain boundary prediction. Annually, most of the well known domain predictors participate in the Critical Assessment of Techniques for Protein Structure Prediction (CASP) competition. Further information on the available datasets can be found at . The dataset contains 66 1-domain chains, 26 2-domain chains and 2 3-domain chains. For each chain we obtained secondary structure information, solvent accessibility, PSSM and Inter-domain linker index by using the previously mentioned methods.

The two raw datasets, Benchmark_2 and CASP7 which contain secondary structure information, solvent accessibility, PSSM and inter-domain linker index were all normalised to fall in the interval [-1, 1] by using following algorithm.

pn = 2*(p-minp)/(maxp-minp) - 1

where

p = R × Q matrix of input vectors,

minp = R × 1 vector containing minimums for each p,

maxp = R × 1 vector containing maximums for each p.

### Proposed model

Protein sequence data can be mathematically viewed as points in a high dimensional space. For example, a sequence of 10 amino acids represents a search space of 20^10 ^possibilities and requires a network of 200 inputs [36]. Learning in the high dimensional space raises several important problems. For example, the large network training requires a large dataset of known examples, which leads to an exponential rise in computational complexity and susceptibility to the overfitting problem (models obtained from high dimensional data fit the training data very well but perform poorly on previously unseen data).

A good dimensionality method eliminates noise or less-discriminatory features that can impede recognition, leaving only a subset of the original features which retain sufficient information to discriminate well among classes. Thus, the number of features used in classification can be reduced when maintaining acceptable classification accuracy. The performance of the most non-parametric statistical classifiers can be extremely dependent upon their input data.

With ANNs, better generalisation and faster training (computationally efficient) can be achieved when they have fewer weights to adjust by fewer inputs. In other words, beyond a certain point, adding new features can actually lead to a reduction in the performance of the classification system [37]. Hence, with a suitable amount of the relevant features, classifiers can not only improve classification speed and efficiency, but also achieve higher accuracy reducing estimation errors associated with finite sample size effects [38].

One solution to the problems above can be semi-parametric modelling. Semi-parametric models take assumptions that are stronger than those of non-parametric models, but, are less restrictive than those of parametric model. In particular, they avoid most serious practical disadvantages of non-parametric methods but at the price of an increased risk of specification errors. The proposed model, Improved General Regression Network (IGRN) takes a form of the semi-parametric model and it finds the optimal trade-off between parametric and non-parametric models. So, it can have advantages of both models while effectively avoiding the curse of dimensionality.

The IGRN contains the evolutionary information compactly represented within the local model. Its global model also works as a collaborative filter that transfers the knowledge amongst local models in formats of hyper-parameters. The local model contains widely known linear principal components. As a collaborative filter, we use General Regression Neural Network. In the literature, GRNN was shown to be effective in noisy environments as it deals with sparse data effectively. Generally, it provides more accurate predictive performance than other existing neural network models. A standard version of the GRNN equation is as follows:

$$\hat{y}(x)=\frac{\sum_{i=1}^{NV}y_{i}\exp\frac{-{\left(x-x_{i}\right)}^{T}(x-x_{i})}{2\sigma^{2}}}{\sum_{i=1}^{NV}\exp\frac{-{\left(x-x_{i}\right)}^{T}(x-x_{i})}{2\sigma^{2}}}$$

where

*x*_*i *_= training vector for class *i *in the input space,

*σ *= single learning or smoothing parameter chosen during network training,

*y*_*i *_= scalar output related to ${\underline{x}}_{i}$, and

*NV *= total number of training vectors.

**Figure 3:**
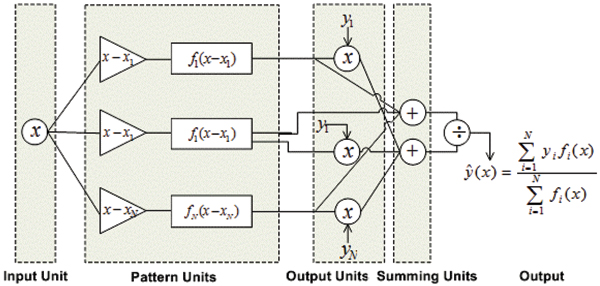
A Basic architecture of GRNN where *f*_*i*_(*x*) represents an arbitrary radial basis function.

Figure 3 shows the most general GRNN architecture where *f*_*i*_(*x*) represents an arbitrary radial basis function. The above equation is represented using a Gaussian radial basis function *f*_*i*_(*x*) as defined by the following equation.

$$f_{i}(x)=\exp\frac{-{\left(x-x_{i}\right)}^{T}(x-x_{i})}{2\sigma^{2}}$$

There are many other radial basis functions that can be used instead of the Gaussian function; however, the radial basis function is commonly chosen due to its computational consideration.

As GRNN is known as a universal approximator for smooth function, it should have the capability to solve any smooth function approximation problems when enough data are provided. However, one drawback is that like general kernel methods, it suffers from the curse of dimensionality. It is unable to ignore irrelevant inputs without major modifications to the basic algorithm. Another major issue with GRNN is its large computational requirements. In the above equation, it incorporates every training vector pair {*x*_*i *_→ *y*_*i*_} into its architecture, (${\underline{x}}_{i}$ is a single training vector in the input space, and *y*_*i *_is the associated desired scalar output). This can require a very large memory space and computations. The two above mentioned issues can significantly degrade the performance of learner when it is applied to high dimensional protein data.

Our method which embeds an efficient linear dimensionality reduction method into the GRNN leads to a more effective approach. If we assume that for each local region of the input space can be represented by reduced vectors generated by an efficient dimensionality reduction method and there is a corresponding scalar output *y*_*i *_into which it maps; then the GRNN can be approximated within acceptable accuracy. This approach not only achieves the network reduction by efficiently reducing the dimensions of input vectors but also creates a more compact network. This method is theoretically proven to be effective for high dimensionality problems dealing with protein data. The general algorithm for IGRN is:

$$\hat{y}(x)=\frac{\sum_{i=0}^{NI}Z_{i}y_{i}\exp\frac{-{\left(x-p_{i}\right)}^{T}(x-p_{i})}{2\sigma^{2}}}{\sum_{i=0}^{NI}Z_{i}\exp\frac{-{\left(x-p_{i}\right)}^{T}(x-p_{i})}{2\sigma^{2}}}$$

where

*x *= input vector,

*p*_*i *_= transformed input vector for class *i *in the input space,

*σ *= single learning or smoothing parameter chosen during network training

*y*_*i *_= scalar output related to *x*_*i*_,

*Z*_*i *_= no. of vectors *x*_*i *_associated with each *p*_*i*_,

*NI *= number of unique transformed input vectors *p*_*i*_.

GRNN is considered as an overfitting and purely non-parametric model, whereas IGRN can be considered as semi-parametric model as it contains the evolutionary information represented with the local model. Hence, the performance of proposed model has some advantages in comparison to the pure parametric models and pure non-parametric models in terms of learning bias and generalisation variance especially on high dimensional protein datasets.

In order to maximise the performance of IGRN, we utilise a network boosting method called Adaptive Boosting (AdaBoost). In general, boosting is known as a technique to improve the performance of any base machine learning algorithms. The AdaBoost algorithm was proposed by Freund and Schapire [39] and it was shown to be a solution for many practical difficulties of previous boosting algorithms. Boosting combines weak learners to find a highly accurate classifier or better fit for the training set [40]. In this study, the AdaBoost was modified for the IGRN for the network boosting. As observed in our experiments, the modified AdaBoost was tested with the IGRN and showed that it could fit into its architecture for the more accurate delineation of domain boundaries. A standard boosting algorithm can be written as:

Given: (*x*_1_, *y*_1_),...,(*x*_*NV*_, *y*_*NV*_) where *x*_*i *_∈ *X*, *y*_*i *_∈ *Y *= {-1, +1}

Initialise *D*_1_(*i*) = 1/*NV*

For *t *= 1,...,*T*:

- Train weak learner using distribution *D*_*t*_

- Get weak hypothesis *h*_*t *_: *X *→ *R*

- Choose *a*_*t *_∈ *R*

- Update:

$$D_{t+1}(i)=\frac{D_{t}(i)\exp(-a_{t}y_{i}h_{t}(x_{i}))}{Z_{t}}$$

where

*NV *= total number of training vectors,

*X *= a domain or instance space of each *x*_*i *_belong to,

*Y *= a label set of each label *y*_*i*_

*Z*_*t *_= a normalisation factor (chosen so that *D*_*t*+1 _will be a distribution),

*R *= its sign is the predicted label {-1, +1}.

Output the final hypothesis:

$$H(x)=sign\left(\sum_{t=1}^{T}a_{t}h_{t}(x)\right)$$

The boosting algorithm takes the training set (*x*_1_, *y*_1_),...,(*x*_*NV*_, *y*_*NV*_) where each *x*_*i *_belongs to a domain *X*, and each label *y*_*i *_is in a label set *Y*. Here, the *Y *should be {-1, +1} as domain boundaries are indicated as positive (+1) or negative (-1) values only. AdaBoost calls the base learning algorithm (IGRN) repeatedly in a series of rounds *t *= 1,...,*T*. And it maintains a distribution or set of weights over the training set. The weight of this distribution in training example *i *in round *t *is denoted *D*_*t*_(*i*). All weights are set equally in the initial state, but on each round, the weights of incorrectly classified examples are increased so that the learner is forced to focus on the hard examples in the training set. The weak learner's job is to find a weak hypothesis *h*_*t *_: *X *→ {-1, +1} appropriate for the distribution *D*_*t*_. The goodness of a weak hypothesis is measured by its error:

$$err_{t}={\mathrm{Pr}}_{i\sim D_{t}}[h_{t}(x_{i})\neq y_{i}]=\sum_{i:h_{t}(x_{i})\neq y_{i}}D_{t}(i)$$

In above equation, the error is measured with respect to distribution *D*_*t *_on which the learner was trained. In some cases where a learner cannot use the weights *D*_*t *_in the training examples, a subset of the training examples can be sampled according to *D*_*t*_, and these unweighted examples can be used to train the learner. In order to validate the effectiveness of the boosting algorithm in the experiments, we IGRN II will indicate AdaBoost embedded in IGRN.

### Other machine learning models

In this study, we also compared the performance of the proposed model with other popular neural network models namely Multi-Layered Perceptron (MLP) and Radial Basis Neural Network (RBFN) and General Regression Neural Network (GRNN). Here, we look at some basic theory of these models and discuss some general issues.

Feed Forward Multi-Layered Perceptron is the most prominent network architecture and primarily used for general classification and regression, implementing feed-forward, supervised and hetero-associative paradigm. In MLP, all nodes and layers are arranged in a feed-forward manner and its signals travels from input layer to output layer only while FBNN allow signals travelling in both directions [41]. The most general feed-forward equation of a three layer Multi-Layered Perceptron (MLP) is as follows:

*y *= *f*_2_(*w*_2_*f*_1_(*w*_1_*x*))

where

*x *= (*x*_1_, *x*_*x*_,...,*x*_3_), a vector of *n *predictive or attribute variables,

*y *= output vector,

*w*_1 _= matrix of linking weights from input to hidden layer,

*w*_2 _= matrix of liking weight from hidden to output layer,

*f*_1 _= transfer function for hidden node,

*f*_2 _= transfer function for output node.

The most popular choice for *f*_1 _and *f*_2 _is the sigmoid function:

*f*_1_(*x*) = *f*_2_(*x*) = (1 + e^-*x*^)^-1^

The aim of training of network is to estimate the weight metrics in the above general MLP equation such that an overall error measure such as the mean squared errors (MSE) or sum of squared errors (SSE) is minimised. MSE can be defined as

$$MSE=\frac{1}{N}\sum_{j=1}^{N}{\left(a_{j}-y_{j}\right)}^{2}$$

where

*a*_*j *_= target value for *j*^*th *^training pattern,

*y*_*j *_= network output for *j*^*th *^training pattern,

*N *= number of training patterns.

MLPs had long been a dominating method in various bioinformatics applications such as prediction of secondary structure, tertiary prediction, domain boundary prediction and so forth.

Like MLP, RBFN is also multi-layer feed-forward network; however, it contains only one hidden layer and the signal in this network is passed forward only. In recent years, RBFN has been widely used due to its structural simplicity and training efficiency.

RBFN combines two different types of learning: supervised and unsupervised. First, at the hidden layer training, one conjoins the input vector into several clusters (unsupervised learning). Afterward, at the output layer training, the output of the RBF network is determined by supervised learning. While for the supervised learning, we have both independent variables and response variables, the unsupervised learning must work without the knowledge of response variables.

RBFN has radial basis functions in hidden nodes and linear functions in output nodes, with adjustable weights that exist only between the hidden and output layers. The output of the *j*^*th *^hidden neuron can be written as:

$$h_{j}=\frac{\varphi (\left\|X-C_{j}\right\|)}{\sigma_{j}}$$

where

*h*_*j *_= output of the *j*^*th *^neuron,

*ϕ *= non-linear radial basis function,

*X *= input vector,

*C*_*j *_= centre of the neuron,

*σ*_*j *_= centre spread parameter.

RBFN builds a local approximation by using radial basis functions that are exponentially decaying nonlinear functions while Feed Forward MLP show a global approximation of nonlinear mapping. In general, RBFN was shown to be a good approach for interpolating scattered data and have been applied in various fields. In RBFN, all input spaces are expressed by the overlapping of receptive fields. It consists of three layers, referred to as input, hidden and output layers. The hidden layer contains several Radial Basis Functions (RBFs) and the Gaussian basis function is the most frequently used RBFs [42].

$$h_{j}(x^{\prime },c_{j},\sigma_{j})=\exp\left[-{\left(\frac{\left\|x-c_{j}\right\|}{\sqrt{2}\sigma_{j}}\right)}^{2}\right]$$

where

||*x *- *c*_*j*_|| = Euclidean distance between an input vector *x *and a centre *c*_*j*_,

*σ*_*j *_= the spread of the *j*^*th *^basis function.

In addition to the Gaussian basis functions, other functional forms can be used for the radial basis functions including some types of thin-plate spline functions, multi-quadratic functions and sigmoidal functions [42]. Any function can be represented in two ways, either as the weighted sum or weighted average. The weighted sum can be presented as:

$$y_{k}(x)=\sum_{j=1}^{m}w_{jk}h_{j}(x)$$

where

*y*_*k*_(*x*) = the *k*^*th *^output,

*w*_*jk *_= weights between the hidden and output layers,

*h*_*j*_(*x*) = output of the *j*^*th *^hidden unit.

The weighted average can be presented as:

$$y_{k}(x)=\frac{\sum_{j=1}^{m}w_{jk}h_{j}(x)}{\sum_{j=1}^{m}h_{j}(x)}$$

It has a higher degree of computational complexity; however, shows better interpolation properties than the weighted sum [43]. The sum of squared error (SSE) between the true values and approximated values from a function is:

$$E=\sum_{p}E_{p}=\sum_{p}\sum_{k}{\left(t_{k}^{\left(p\right)}-y_{k}^{\left(p\right)}\right)}^{2}$$

which is the sum of residuals of each observation (or sample).

RBFNs usually have three kinds of parameters: the centre and weight of each radial basis function and the weights between the hidden and output layers. Thus, fast and precise identification of these parameters is of primary concern. The centre and width are nonlinear parameters, whereas the weight is linear. Generally, two learning strategies have been applied to RBFNs, nonlinear training methods and hybrid learning methods.

### Training, testing and validation

In order to thoroughly test the performance of our proposed model, we conducted two extensive experiments on two benchmark datasets. For the experiment on the Bechmark_2 dataset, we adopted a cross-validation scheme for the model evaluation. As with jittering, cross-validation is regarded as one of the effective approaches to improve generalisation of a model. We adopted seven fold cross validation method and random data set selection and testing was conducted seven times for each window size dataset. When multiple random training and testing experiments were performed, a model was formed from each training sample. The estimated prediction accuracy is the average of the prediction accuracy for the models and each window size, derived from the independently and randomly generated test divisions. If the predicted domain boundary is in the range of ±15 residues of the true domain boundary, we consider it as a correct prediction [18]. We tested four different window sizes (7, 11, 19 and 27) for each model and window size 7 showed the best accuracy for all predictors.

During the post-processing of the network output, as the network generates the raw outputs which have many local peaks, we filtered these raw outputs by using Liu and Rost's [27] method. We determined the threshold for each network outputs according to the length (*L*) of the protein and to the distribution of raw output values for all residues in that protein. We compiled the 92nd percentile of the raw output *T1 *and set the threshold *T *to

$$T=\{\begin{array}{ll} \max(T_{1},60) & for\;L\leq 100\\ \max(T_{1},30) & for\;L\leq 200\\ T_{1} & for\;L>200 \end{array}$$

*T *was set to the threshold that divides domain boundaries and others. If the value of a residue is above the threshold, the residue is regarded as domain boundary. Second, we assigned the central residue as a domain boundary if three or more residues were predicted as a domain boundary. And all parameters for these filters were developed using the validation set only.

As for the experiment on CASP7 dataset, we trained each network using the window size 7 training sets obtained from the experiment on the Benchmark_2 dataset. Each protein chain in CASP7 dataset was fed into each network and a prediction was made. Also identical post-processing procedure was conducted as performed in the experiment on the Benchmark_2 dataset. With the CASP7 dataset, if the predicted domain boundary is in the range of ±15 residues of the true domain boundary (CATH assigned dbp) then we consider it as a correct prediction.

The stepwise procedure we have performed can be summarised as follows:

(1) Data collection, building a new dataset and pre-processing datasets.

(2) Obtain protein structural information such as PSSM, solvent accessibility, secondary structure and linker index by utilising PSI-BLAST [21], ACCpro [34], SSpro [35] and DomCut [16].

(3) The information obtained in (2) was combined and normalised to fall in the interval [-1, 1] to be fed into networks.

(4) Assign target levels: positive (1) for boundary residues and negative (-1) for non-boundary residues.

(5) Hold-out method was performed to divide the combined dataset into 7 subsets (training and testing sets).

(6) Train each model on training set to create a model.

(7) Simulate each model on test set to obtain predicted outputs.

(8) Post-processing was performed to find predicted boundary locations.

## List of abbreviations used

ANN: Artificial Neural Network

GRNN: General Regression Neural Network

IGRN: Improved General Regression Network

MLP: Multi-Layered Perceptron

PSI-BLAST Position Specific Iterated Basic Local Alignment Search Tool

PSSM: Position Specific Scoring Matrix

RBFN: Radial Basis Function Neural Network

## Competing interests

The authors declare that they have no competing interests.

## Authors' contributions

PDY developed and implemented the new model (IGRN) and drafted the manuscript. ARS prepared datasets, programming in C++, Shell and Perl scripts, and interpreted the results with PDY. BBZ and AYZ edited the manuscript and introduced the problem initially.
